# Influence of Long-Lasting Static Stretching on Maximal Strength, Muscle Thickness and Flexibility

**DOI:** 10.3389/fphys.2022.878955

**Published:** 2022-05-25

**Authors:** Konstantin Warneke, Anna Brinkmann, Martin Hillebrecht, Stephan Schiemann

**Affiliations:** ^1^ Department for Exercise, Sport and Health, Leuphana University, Lüneburg, Germany; ^2^ Assistive Systems and Medical Device Technology, Carl von Ossietzky University of Oldenburg, Oldenburg, Germany; ^3^ University Sports Center, Carl von Ossietzky University of Oldenburg, Oldenburg, Germany

**Keywords:** static stretching, muscle cross sectional area, maximal strength, range of motion, hypertrophy

## Abstract

**Background:** In animal studies long-term stretching interventions up to several hours per day have shown large increases in muscle mass as well as maximal strength. The aim of this study was to investigate the effects of a long-term stretching on maximal strength, muscle cross sectional area (MCSA) and range of motion (ROM) in humans.

**Methods:** 52 subjects were divided into an Intervention group (IG, *n* = 27) and a control group (CG, *n* = 25). IG stretched the plantar flexors for one hour per day for six weeks using an orthosis. Stretching was performed on one leg only to investigate the contralateral force transfer. Maximal isometric strength (MIS) and 1RM were both measured in extended knee joint. Furthermore, we investigated the MCSA of IG in the lateral head of the gastrocnemius (LG) using sonography. Additionally, ROM in the upper ankle was investigated *via* the functional “knee to wall stretch” test (KtW) and a goniometer device on the orthosis. A two-way ANOVA was performed in data analysis, using the Scheffé Test as post-hoc test.

**Results:** There were high time-effects (*p* = 0.003, ƞ² = 0.090) and high interaction-effect (*p* < 0.001, ƞ²=0.387) for MIS and also high time-effects (*p* < 0.001, ƞ²=0.193) and interaction-effects (*p* < 0.001, ƞ²=0,362) for 1RM testing. Furthermore, we measured a significant increase of 15.2% in MCSA of LG with high time-effect (*p* < 0.001, ƞ²=0.545) and high interaction-effect (*p*=0.015, ƞ²=0.406). In ROM we found in both tests significant increases up to 27.3% with moderate time-effect (*p* < 0.001, ƞ²=0.129) and high interaction-effect (*p* < 0.001, ƞ²=0.199). Additionally, we measured significant contralateral force transfers in maximal strength tests of 11.4% (*p* < 0.001) in 1RM test and 1.4% (*p*=0.462) in MIS test. Overall, there we no significant effects in control situations for any parameter (CG and non-intervened leg of IG).

**Discussion:** We hypothesize stretching-induced muscle damage comparable to effects of mechanical load of strength training, that led to hypertrophy and thus to an increase in maximal strength. Increases in ROM could be attributed to longitudinal hypertrophy effects, e.g., increase in serial sarcomeres. Measured cross-education effects could be explained by central neural adaptations due to stimulation of the stretched muscles.

## Introduction

Regular stretch training over several weeks improves flexibility and range of motion (ROM) (Young et al., 2013; Medeiros et al., 2016). Reduced pain perception due to habituation effects in humans (Freitas et al., 2018) and muscle fiber lengthening due to serial accumulation of sarcomeres following intensive stretch training could be determined in animals, which could be responsible for enhanced flexibility (Williams et al., 1988; Alway S. E., 1994). A transfer to human training studies can be hypothesized, as Damas et al. (2018) demonstrated increased serial sarcomere accumulation in humans in general. To maximize ROM, stretch training should include a long stretching duration with a high training frequency (Thomas et al., 2018). In addition to stretching duration and frequency, stretch intensity has a crucial influence on muscular adaptations. At low stretching intensities, it can be assumed that the tension is compensated primarily by the elastic components so that effects on the contractile tissue are only achieved at a certain minimum intensity (Apostolopoulos et al., 2015).

Long-term stretching of a muscle can also increases muscle mass due to muscular hypertrophy in animals. A variety of studies have been investigated in birds for this purpose, in which a wing of the test animal was stretched from 30 min daily to a 24-h continuous stretch over a period of 1 month (Frankeny et al., 1983; Williams et al., 1988; Antonio et al., 1993; Alway S. E., 1994; Czerwinski et al., 1994). In animal examination, Antonio & Gonyea (1993) achieved an enhancement in muscle mass of 318% with an intermittent stretching protocol by increasing stretching intensity from 10% of the bodyweight to 25% over 33 days. Stretching one wing of quails and chickens for different stretching durations demonstrated an increase in muscle mass depending on stretching duration (Bates, 1993; Frankeny et al., 1983; J.; Lee & Alway, 1996). Furthermore, gains in muscle mass in listed studies can be related to longitudinal hypertrophy and increases in muscle cross-sectional area of over 100% (Frankeny et al., 1983; Matthews et al., 1990; Alway S. E., 1994). Improvements in maximal strength are often related to enhanced muscle cross sectional area (Seitz et al., 2016). In quail, Alway S. E., 1994 found increments of maximal strength of 95% by continuous stretching for 30 days compared to the contralateral control muscle by *in vitro* studies.

Since authors investigated significant muscular hypertrophy in quail and chicken wings due to long lasting stretching interventions of several hours, which are in correlation with improvements in maximal strength (Alway S. E., 1994), question arises whether effects in maximal strength as well as in muscle cross-sectional area are transferable to humans. In a meta-analysis, Medeiros & Lima (2017) determined a positive effect of stretching on muscular performance measured *via* functional tests and isotonic contractions in humans. In addition, literature shows significant improvements in maximal strength up to 32.4% in leg extension by stretching the lower extremity. For this, a 40-min stretching workout was performed three times per week which was divided into 15 different stretching exercises for lower extremities, each hold for 3 × 15 s (Kokkonen et al., 2007). Highest stretching duration was performed by (Yahata et al., 2021) by stretching the plantar flexors with a specific stretching board for 30 min per session, each session twice a week for 5 weeks. While Yahata et al. (2021) reported improvements in maximal strength of 6.4%, Mizuno (2019) showed increases in maximal strength of 20.2% in maximal strength with a stretching intervention for 8 weeks. However, other studies failed to point out any significant changes in MCSA or maximal strength after several weeks of stretching training (Sato et al., 2020; Longo et al., 2021; Nakamura et al., 2021).

Furthermore, Panidi et al. (2021) and Kokkonen et al. (2007) demonstrated improvements in jumping performance of up to 22% (Panidi et al., 2021). While Nunes et al. (2020) point out that low intensity stretching intervention is not a sufficient stimulus to induce muscular hypertrophy, Panidi et al. (2021) examined an enhancement in muscle thickness of 23% due to a stretching training for 12 weeks in volleyball players. Moreover, Simpson et al. (2017) showed increments of 5.6% in muscle thickness due to 3 minutes stretching stimulus on 5 days a week.

In addition to improved maximal strength of 29% in the stretched leg, Nelson et al. (2012) showed significant increases in maximal strength in the contralateral leg of 8%. Panidi et al. (2021) also point out contralateral improvements in muscular performance measured in unilateral CMJ. To this point, cross-education effects are mostly known from strength training when conducted unilaterally (Andrushko et al., 2018a; Andrushko et al., 2018b; M.; Lee et al., 2009; Lee & Carroll, 2007). We were not able to find other studies investigating long-term effects of stretching durations lasting at least 1 hour per day on maximal strength as well as muscle thickness.

Consequently, no statement about transferability of results from animal studies can be given, so the aim of the present work is to investigate the adaptive responses to a daily one-hour stretching training in maximal strength, muscle cross-sectional area as well as ROM. In addition, single-leg stretching is used to investigate cross education effects by using the non-stretched leg as an intra-inidividual control condition. We hypothesize, that 1 hour of stretching over 6 weeks lead to enhanced maximal strength, muscle thickness and ROM in the stretched leg. Furthermore, we suggest improvements in maximal strength in the not intervened control leg.

## Methods

### Subjects

G-Power analysis was performed to estimate the required sample size showing a minimal total sample size of 36. 52 athletically active subjects were recruited from sports study programs, sports clubs, and fitness studios. Participants were classified as active athletes if they performed two or more training sessions per week in a gym or a team sport continuously for the previous 6 months. Subjects performing daily stretching training or similar activities like yoga as well as untrained subjects were excluded from the study. Included subjects were randomly divided into an intervention group (IG) and a control group (CG). One participant was dropped out, because of a sports related injury of his knee joint. Characteristics of subjects are displayed in Table 1.

**TABLE 1:** Characteristics of test subjects.

Group	N	Age (in years)	Height (in cm)	Weight (in kg)
Total	52 (f = 21, m = 31)	27.0 ± 3.1	175.9 ± 5.2	80.5 ± 7.3
IG	27 (f = 11, m = 16)	27.4 ± 3.1	176.2 ± 5.6	81.0 ± 6.2
CG	25 (f = 10, m = 15)	26.8 ± 2.9	175.6 ± 4.9	79.3 ± 5.3

All participants were informed about the experimental risks and provided written informed consent to participate in the present study. Furthermore, approval for this study was obtained from the institutional review board (Carl von Ossietzky Universität Oldenburg, No.121-2021). The study was performed with human participants in accordance with the Helsinki Declaration.

### Intervention

**FIGURE 1:**
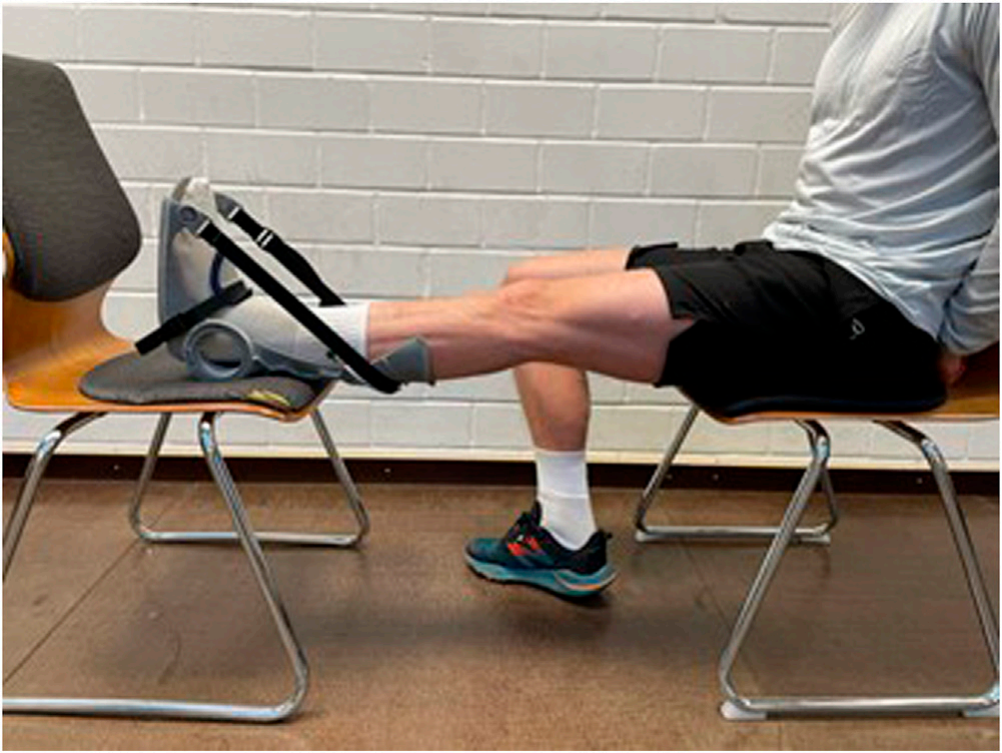
Orthosis used for calf muscle stretching.

The intervention consisted of daily stretching training of the calf muscles lasting 1 hour each session for 6 weeks, which was realized by wearing an orthosis designed for this purpose (Figure 1). The intervention was performed with the dominant leg only to give the opportunity to evaluate potential cross-sectional effects. To define the dominant leg, participants were asks about which leg they use when perform single-leg jumps. Subjects were instructed to wear the orthosis with extended knee joint and the stretch Intensity was controlled by an goniometer which was also used to determine the angle representing the starting value during the pre-test. To achieve high intensity and muscle tension during the stretching training, subjects were asked to reach maximal dorsiflexed position with an individual stretching pain at eight on a scale 1 to 10. The angle of the orthosis had to be set on corresponding angle to ensure sufficient intensity. Consequently, set angle of the orthosis should improve with enhanced ROM. The stretching was to be performed 7 days a week in a standardized body posture: the subjects were instructed to sit with their backs as straight as possible and place their feet on a support plate at the same height as their chair. All subjects in the intervention group borrowed one orthosis for the duration of the intervention and had to complete a stretching diary in which the daily stretching duration as well as the set angle were written down to record stretching duration and intensity. The control group did not perform any stretching interventions.

### Testing Procedure

Before testing a five-minute warm up routine consisting of 5 min with a 130-bpm heart rate ergometer cycling was performed.

#### Maximal Strength Measurement

**FIGURE 2:**
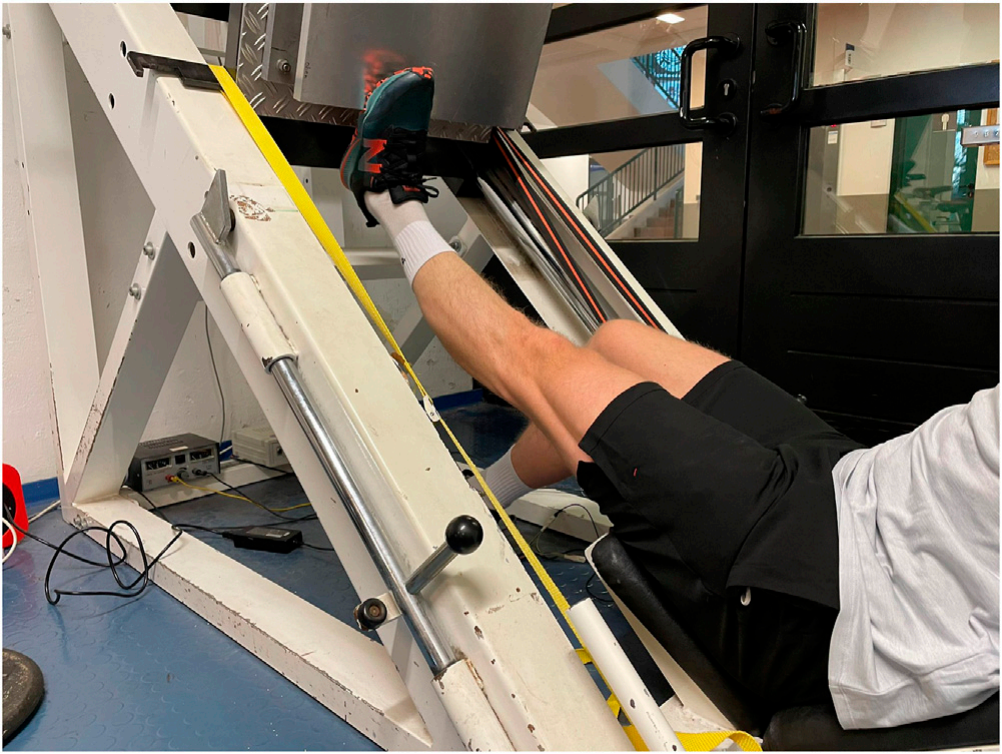
Testing device for maximal isometric strength in extended knee using leg press (LP).

All subjects participated in the pre- and post-test. Maximal isometric and dynamic strength were assessed using single-leg testing in extended as well as in flexed knee joint. A 45° leg press was used to measure maximal strength in the extended knee joint. A force plate was attached to the footpad to record the maximal strength in the calf muscles with extended knee joint. We used an 50 × 60 cm force plate with a measuring range of ± 5000N and a 13-bit analog-to-digital converter. To measure maximal isometric strength, the subject was instructed to place the feet on the attached force plate such as that the metatarsophalangeal joints of the feet were placed on the edge flush (Figure 2). The starting position was chosen to give a 90° ankle joint angle, which was controlled *via* the placement of an angle template. The force plate was fixed to form an impassable resistance from this position. The subject was instructed to perform a maximal voluntary contraction with a plantarflexion in response to an audible signal. Participants had to hold maximal contraction for at least one second after reaching perceived maximal strength. Force-time curve was recorded for 5 s. After each trial, a one-minute rest was observed to avoid fatigue. Measurements were conducted until no improvement in maximal strength was recorded but for a minimum of three trials. Reliability was determined between best trial and second-best trial, for which a high reliability can be considered Table 2. In the following, after taking a recovery break of 5 min, the maximal dynamic strength of the calf muscles was tested with the knee joint extended. The subject was instructed to assume the starting position (90° ankle joint angle) and to press the applied weight into a maximal plantarflexed position. For this purpose, the covered distance was recorded with a motion sensor from the company “MicroEpsilon” with an accuracy of 0.1 mm. Based on isometric data of the previous testing, we added weight corresponding to 60% of the maximal strength. After each trial, we added weight (first 10 kg, then 5 or 2.5 kg) on the leg press until the participant was no longer able to perform the 1RM for full ROM. The criterion for the end of measurement was the distance measurement *via* the motion sensor. Best trial with full ROM measured was used for further analysis.

**TABLE 2:** Reliability for the pre-test values. ICC = intraclass correlation coefficient, CV = coefficient of variance, SD = Standard deviation.

Parameter	ICC	CV (%)	SD
LPisoil	0.954	1.68	24.29
LPisocl	0.971	1.82	25.58
LPisoCGR	0.968	2.21	35.28
LPisoCGL	0.964	1.83	27.27
SONOil	0.947	2.99	4.6
SONOcl	0.971	1.93	7.07
KtWil	0.987	1.74	0.21
KtWcl	0.992	0.94	0.13
KtWCGR	0.979	1.81	0.24
KtWCGL	0.991	1.40	0.16
ORTil	0.997	0.64	0.38
ORTcl	0.997	0.62	0.38
ORTCGR	0.989	0.78	0.7
ORTCGL	0.990	1.16	0.8

LP, leg press; iso, isometric maximal strength; il, intervened leg; cl, control leg; Wt, weight in dynamic maximal strength; CG, control group; R, right; L, left.

#### Measuring Muscle Thickness

**FIGURE 3:**
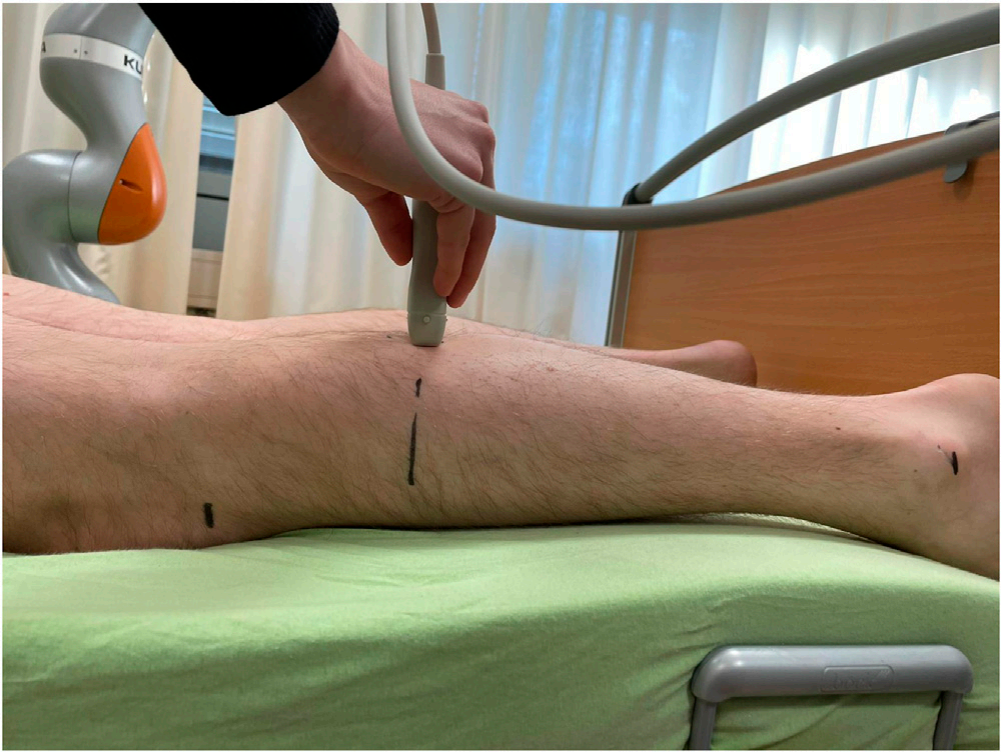
Sonography to investigate muscle thickness in the calf muscle.

Measures of skeletal muscle architecture were done using two-dimensional B-mode ultrasound (Mindray Diagnostic Ultrasound System). Here, muscle thickness represents the most employed measure of muscle dimension (Sarto et al., 2021) according to its correlation to muscle cross-sectional area, which is proportional to the number of parallel sarcomeres, thereby influencing maximal force production (Lieber & Fridén, 2000; Narici et al., 2016; May et al., 2021). In our examination, ultrasound images from the lateral gastrocnemius were recorded using a linear transducer with a standardized frequency of 12–13 MHz. Each participant was placed prone on a table with the feet hanging down at the end to ensure no contraction in the calf muscles. Then, the sonographer identified the proximal and distal landmark of the lateral gastrocnemius for each participant and measurement (Perkisas et al., 1999). The transducer was placed at 30% of the distance from the most lateral point of the articular cleft of the knee to the most lateral top of the lateral malleolus (see Figure 3) (Perkisas et al., 1999). For measuring muscle thickness, the transducer was positioned at the midpoint of the muscle belly perpendicular to the long axis of the leg (Sarto et al., 2021). The muscle belly was determined as the center of the muscle between its medial and lateral borders. This is the point where the muscle’s anatomical cross-sectional area is maximal (Fukunaga et al., 1992). In addition, the image plane is best aligned with the muscle’s fascicles, including minimal fascicle curvature (Bénard et al., 2009; May et al., 2021; Raj et al., 2012). Before starting the measurement, transmission gel was applied to improve acoustic coupling and to reduce the transducer pressure on the skin. Then, the sonographer ensured that the superficial and deep aponeuroses were as parallel as possible by holding and thereby rotating the transducer around the sagittal-transverse axis to the determined point on the skin without compressing the muscle. Hence, the visibility of the fascicles as continuous striations from one aponeurosis to the other was optimized. Muscle thickness is defined as the linear, perpendicular distance between the two linear borders of the skeletal muscle and was obtained by averaging three measurements across the proximal, central, and distal portions of the acquired ultrasound images (Franchi et al., 2017; Sarto et al., 2021). Two persons independently evaluated muscle thickness using the image processing software GIMP 2.10.28. The objectivity of the evaluators was found to be between 0.85 (control leg) and 0.94 (intervention leg).

In the literature, high-reliability values of up to r = 0.9 for determining muscle thickness *via* ultrasound for within-day reliability (Nabavi et al., 2014; Cuellar et al., 2017) and with ICC values of up to 0.88 for between-day reliability are considered high (König et al., 2014; Rahmani et al., 2019a, 2019b).

Reliability was determined between best and second-best value and the “with-in day” reliability determined in this paper can be classified as high with a value of r = 0.98. ICC, CV and SD are listed in Table 2, too. Two persons evaluated the ultrasound images independently from each other.

### ROM Measurement

**FIGURE 4:**
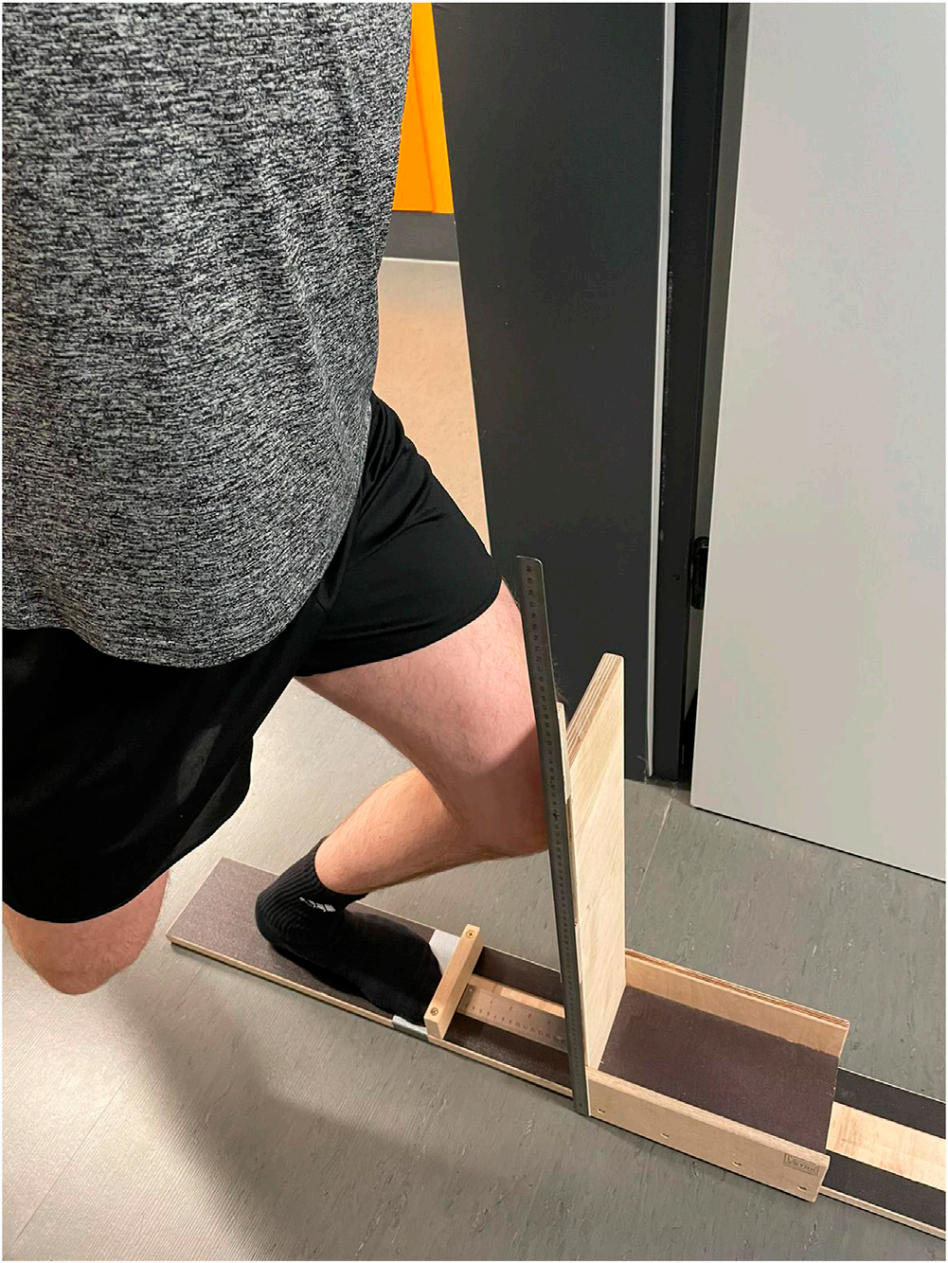
Sliding device for the KtW to evaluate flexibility in the ankle.

ROM in the upper ankle joint was recorded in IG and CG *via* the functional “knee to wall stretch” test (KtW) and the angle-measuring device on the orthosis. A sliding device was used for the KtW. The subject was instructed to place the foot on the attached marker. The contralateral leg was held in the air, and the subject was allowed to hold onto the wall with his hands. To record the range of motion, the subject pushed the board of the sliding device forward until the heel of the standing leg lifted off. For this purpose, the investigator pulled on a sheet of paper placed under the subject’s heel. The measurement was finished as soon as this could be removed. The mobility was read in cm from the attached measuring tape (Figure 4). Depending on ankle ROM, this measurement can be seen as screening flexibility in bended knee. Three valid trials were performed per leg, and the maximal value was used for evaluation. Reliability was determined between best trial and second-best trial and can be classified as high Table 2.

**FIGURE 5:**
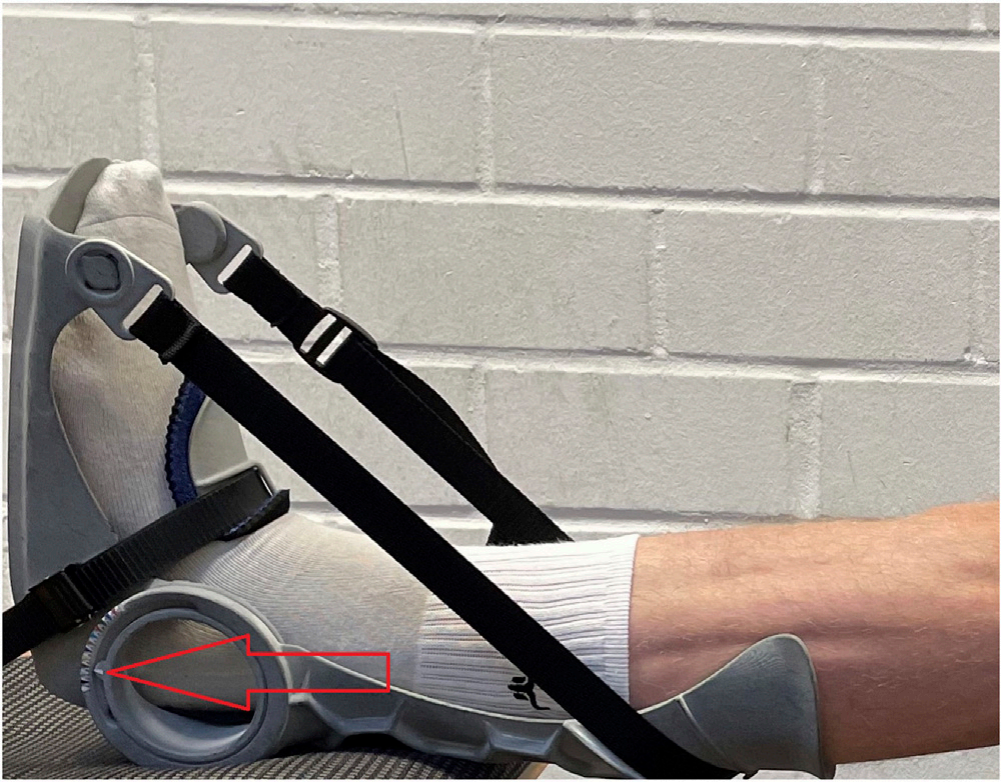
Measuring device for maximal dorsiflexion *via* goniometer attached to the orthosis.

Since we measured maximal strength in extended knee joint, we used the angle measurement device of the orthosis which could be used as goniometer (ORT) to measure maximal dorsiflexion in extended knee joint (see Figure 5). For this purpose, the foot of the participant should place his foot on a support plate at the same height as the chair. While wearing orthosis the foot was pushed into maximal dorsiflexed position with extended knee joint. Starting position was neutral 0 position in the ankle. Each big mark of the angle measurement device corresponds to a distance of 5°, and each little mark corresponds to a distance of 2.5°. The achieved marker was read off from the angle measurement device of the orthosis. Reliability was determined between best trial and second-best trial and can be classified as high, Table 2.

**FIGURE 6:**
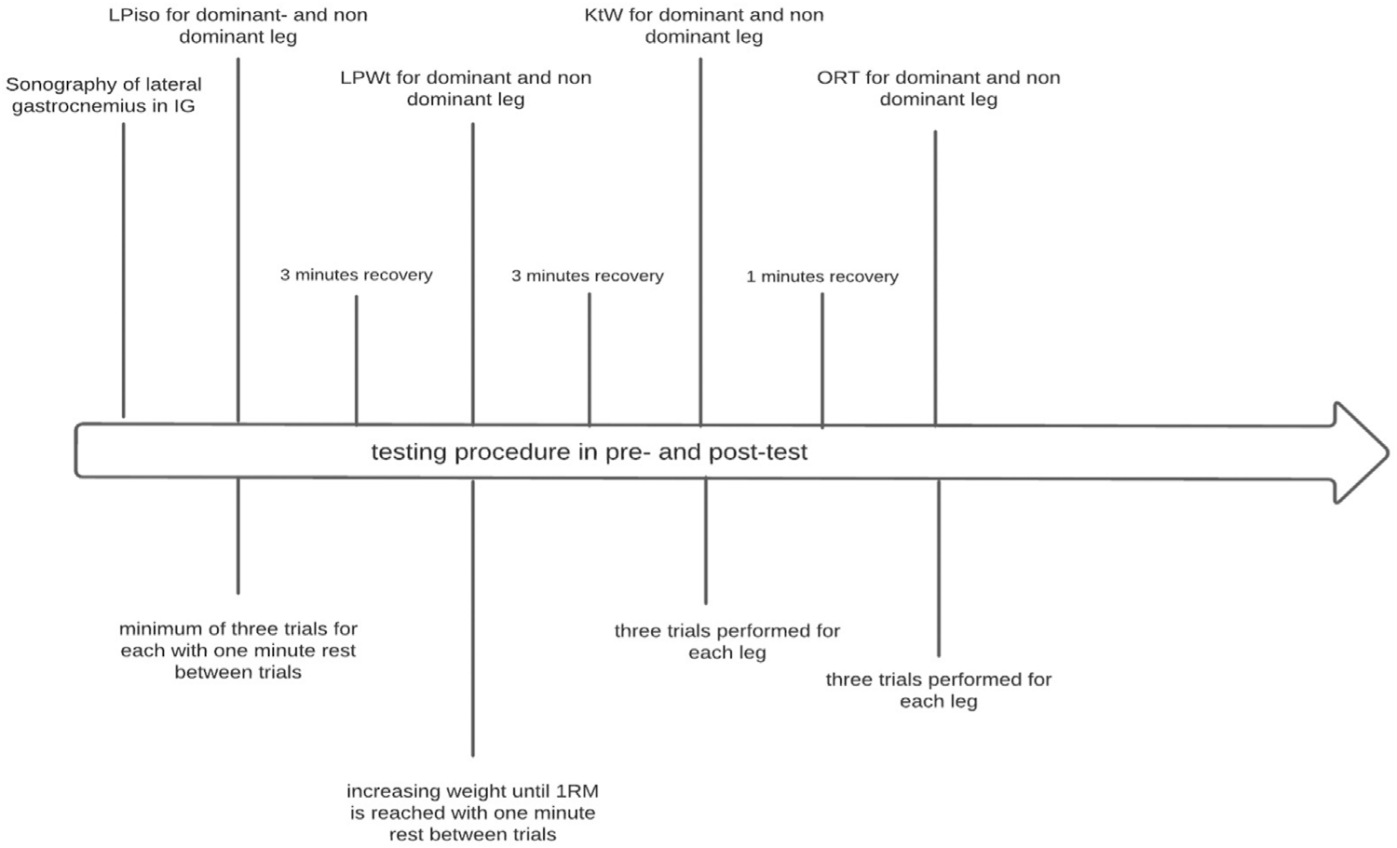
Graphical presentation of study design.

To improve comprehension of testing procedure, in Figure 6 the study design is presented graphically.

### Data Analysis

The analysis was performed with SPSS 28. We used one-way ANOVA with Scheffé post-hoc test to ensure that there were no differences in pre-test values for any measurement. Thus, two-way ANOVA with repeated measures was performed for the collected parameters. Scheffé test was used as post-hoc for mean differences of one-way ANOVA. *p*-Values for percentage changes were determined with pared t-test between pre- and posttest. Effect sizes were presented as Eta squares (ƞ^2^) and categorized as: small effect ƞ^2^<0.06, medium effect ƞ^2^ = 0.06–0.14, large effect ƞ^2^>0.14 as well as Cohen’s d. (Cohen, 1988) Effect sizes with Cohen’s d were categorized as: small effects d < 0.5, medium effect d = 0.5–0.8, large effect d > 0.8. In addition, Pearson correlations were determined between maximal strength and muscle thickness as well as between changes in maximal strength and muscle thickness.

## Results

All subjects who appeared for the pretest completed the examination. No significant problems with the orthosis were reported and the daily wearing durations were adhered to all subjects.

Results of descriptive statistics as well as the two-way ANOVA are presented in Table 3. P- and F- Values of the two-way ANOVA as well as effect sizes ƞ^2^ for time dependent effect and interaction effects are displayed.

**TABLE 3:** Descriptive statistics and two-way ANOVA of maximal strength tests.

Parameter	Pretest (M±SD)	Posttest (M±SD)	Pre-post differences in %	Time effect	Time x group
LPIsoil	1478.4 ± 309.7N	1726.8 ± 315.8N	16.8 (*p* < 0.001)	*p* < 0.003	*p* < 0.001
LPIsocl	1542.3 ± 339.1N	1564.5 ± 300.5N	1.4 (*p* = 0.462)	F = 9.108	F = 19.387
CGR	1585.4 ± 215.1N	1559.0 ± 217.8N	−1.6 (*p* = 0.075)	ƞ^2^ = 0.090	ƞ^2^ = 0.387
CGL	1540.1 ± 184.94N	1518.0 ± 202.55N	−1.4 (*p* = 0.164)	d = 0.629	d = 1.589
LPWtil	91.9 ± 35.0 kg	115.0 ± 32.3 kg	25.1 (*p* < 0.001)	*p* < 0.001	*p* < 0.001
LPWtcl	93.5 ± 32,3 kg	104.2 ± 34.4 kg	11.4 (*p* < 0.001)	F = 22.028	F = 17.434
CGR	96.9 ± 27.6 kg	95.0 ± 28.6 kg	−1.2 (*p* = 0.467)	ƞ^2^ = 0.193	ƞ^2^ = 0.362
CGL	98.6 ± 27.8 kg	95.0 ± 28.4 kg	−3.6 (*p* = 0.214)	d = 0.978	d = 1.506

LP = leg press; iso = isometric maximal strength; il = intervened leg; cl = control leg; Wt = weight in dynamic maximal strength; CG = control group; R = right; L = left.

### Analysis of Maximal Strength With Extended Knee Joint *via* Leg Press

One-way ANOVA showed no significant differences between pretest values of all parameters (*p* > 0.05).

Progression and comparison of mean values of maximal strength in pre- and post-testing in the stretched and the control leg of the intervention group is presented in Supplementary Figure S1.

Two-way ANOVA demonstrated high effects for the time dependent effect (ƞ^2^ = 0.09 and 0.193) and for the time × group interaction (ƞ^2^ = 0.387 and 0.362).

The Scheffé test determined significant differences for the mean differences between pre- and posttest values in the LPisoil and the LPisocl as well as LPisoil and CGR (*p* < 0.001) and LPisoil and CGL (*p* < 0.001). No significant difference could be determined between the control leg and CGR (*p* = 0.415) as well as control leg and CGL (0.812). Between the legs of the CGs, no significant difference could be detected (*p* = 0.927).

For maximal dynamic strength there were significant differences for the mean differences between pre- and posttest values in LPWtil and LPWtcl (*p* = 0.026), LPWtil and CGR (*p* < 0.001), LPWtil and CGL (*p* < 0.001) as well as LPWtcl and CGR (*p* = 0.026) and LPWtcl and CGL (*p* = 0.014). No significant difference could be determined between CGR and CGL (*p* = 0.987).

### Analysis of Muscle Thickness *via* Sonography

Table 4 shows descriptive statistics as well as time dependent effect and interaction effects of tow-way ANOVA for determining muscle thickness in the calf muscle.

**TABLE 4:** Descriptive statistics and two-way ANOVA of muscle thickness *via* sonography.

Parameter	Pretest (M±SD) in mm	Posttest (M±SD) in mm	Pre-post differences in %	Time effect	Time x group
SONOil	14.31 ± 2.42	16.5 ± 2.78	15.3 (*p* < 0.001)	*p* < 0.001	*p* = 0.015
SONOcl	14.54 ± 2.32	14.85 ± 2.08	2.1 (*p* = 0.03)	F = 33.588	F = 19.166
				ƞ^2^ = 0.545	ƞ^2^ = 0.406
				d = 2.189	d = 1.653

SONO, sonography; il, intervened leg cl, control leg.

**FIGURE 7:**
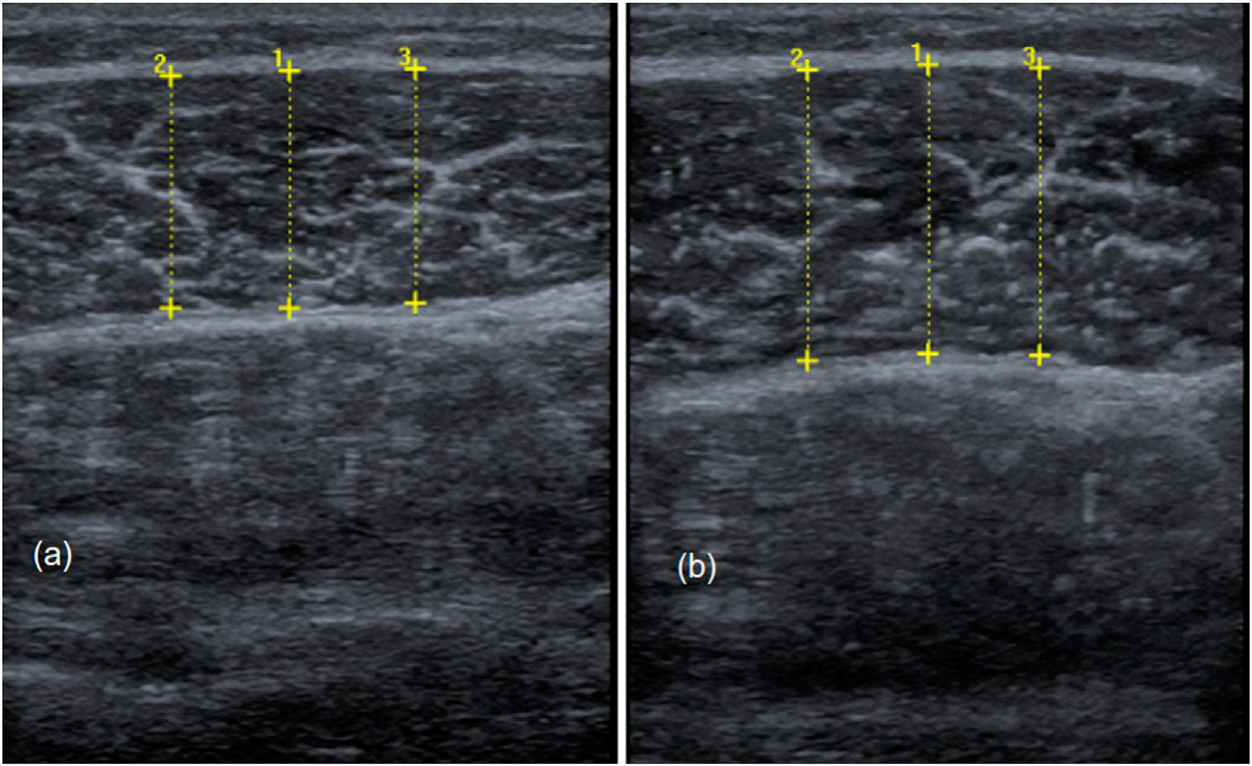
Comparison of muscle thickness from pre-to posttest in the non-stretched control leg **(A)** and the intervened leg **(B)**.

Figure 7 shows examples of sonography measurements from pre to posttest of the control leg and the intervened leg.

Progression and comparison of mean values of muscle thickness in pre- and post-testing in the stretched and the control leg of the intervention group is presented in Supplementary Figure S2.

Two-way ANOVA demonstrated high effects for the time dependent effect (ƞ^2^ = 0.545) and for the time × group interaction (ƞ^2^ = 0.406).

### Analysis of ROM Values

Progression and comparison of mean values of ROM tested *via* KtW and the angle measurement device of the orthosis (ORT) in pre- and post-testing in the stretched and the control leg of the intervention group is presented in Table 5 and in Supplementary Figure S3.

**TABLE 5:** Descriptive statistics and two-way ANOVA of ROM tests.

Parameter	Pretest (M±SD)	Posttest (M±SD)	Pre-post differences in %	Time effect	Time x group
KtWil	12.1 ± 3.0 cm	13.7 ± 2.6 cm	13.2 (*p* < 0.001)	*p* = 0.011	*p* < 0.001
KtWcl	12.7 ± 3.9 cm	12.6 ± 3.7 cm	−0.8 (*p* = 0.701)	F = 6.674	F = 16.925
CGR	12.6 ± 1.1 cm	12.3 ± 2.0 cm	−2.4 (*p* = 0.007)	ƞ^2^ = 0.068	ƞ^2^ = 0.356
CGL	12.2 ± 1.8 cm	12.1 ± 1.5 cm	−0.8 (*p* = 0.506)	d = 0.54	d = 1.487
ORTil	6.7 ± 1.9	8.4 ± 2.0	27.3 (*p* < 0.001)	*p* < 0.001	*p* < 0.001
ORTcl	6.8 ± 1.9	7.2 ± 2.1	7.5 (*p* = 0.211)	F = 13.527	F = 7.613
CGR	7.6 ± 1.4	7.6 ± 1.3	0.7 (*p* = 0.724)	ƞ^2^ = 0.129	ƞ^2^ = 0.199
CGL	7.6 ± 1.6	7.6 ± 1.6	0 (*p* = 1.000)	d = 0.77	d = 0.997

KtW, knee to wall stretch; il, intervened leg; cl, control leg; CG, control group; ORT, angle measuring device of the orthosis; R, right; L, left.

Two-way ANOVA demonstrated high effects for the time dependent effect (ƞ^2^ = 0.068 and 0.129) and for the time × group interaction (ƞ^2^ = 0.356 and 0.199). The Scheffé test determined significant differences for the mean differences between pre-to posttest KtWil and KtWcl (*p* < 0.001) as well as between KtWil and CGR (*p* < 0.001) and CGL (*p* < 0.001). No significant difference was found between the control leg and CGR (*p* = 0.941) and CGL (*p* = 1.000). Furthermore, no significant difference was found between CGR and CGL (*p* = 0.959).

Significant differences were found for the mean differences between pre-to posttest for ORTil and the ORTcl (*p* = 0.019) and ORT and CGR (*p* = 0.002) and CGL (0.002). No significant differences were found between ORTcl and CGR (*p* = 0.838) and CGL (*p* = 0.783), as well as CGR and CGL (*p* = 1.000) measured *via* angle measuring device of the orthosis.

Pearson correlations determined for muscle thickness and maximal strength show correlations of r = 0.594 in the pre-test as well as 0.74 for post-test values. However, Pearson correlation for increases from pre-to post-test show no significant relationship with r = 0.02 (*p* = 0.935).

## Discussion

In previous research, we already compared effects of one hour vs. two hours static stretching on maximal isometric strength in bended knee joint. Significant differences in required muscle groups in maximal strength testing between bended and extended knee joint (Signorile et al., 2002; Arampatzis et al., 2006) as well as type of contraction—isometric vs. dynamic testing condition—(Murphy & Wilson, 1996; Feeler et al., 2010) can be assumed.

In this work, a significant improvement in maximal strength in the calf muscles was achieved by daily one-hour stretching training. There was a significant improvement in maximal isometric strength production determined in the extended knee joint by approximately 16.8% from 1478.4 ± 309.7N in pretest to 1726.8 ± 315.8N in the stretched leg. In comparison, an average maximal strength increase of 1.4% from 1542.3 ± 339.1N to 1564.5 ± 300.5N was determined in the non-stretched control leg while no significant increase was determined between legs of CG. Furthermore, we determined enhanced maximal dynamic strength *via* 1RM testing by 25.1% and 11.4% from 91.9 ± 35 kg to 115 ± 32.3 kg and 93.5 ± 32.3 kg to 104.2 ± 34.4 kg in the stretched and non-stretched control leg, respectively. In both legs in CG no significant change in 1RM could be determined. For all maximum strength measurements, large effect sizes were shown for interaction effect in ANOVA (ƞ^2^>0.14 and d > 0.8). In addition, we measured significant hypertrophy effects in the lateral head of the gastrocnemius of 15.2% from in the intervention leg vs. 2.1% in the control leg. In the intervened leg, we determined and increase 14.31 ± 2.42 mm to 16.5 ± 2.78 mm. In control leg muscle thickness, we found muscle thickness of 14.54 ± 2.32 in pretest and 14.85 ± 2.08 mm in posttest. Furthermore, moderate correlations between maximal strength values in the extended knee joint and muscle thickness in the pre-test (r = 0.594; *p* = 0.012) and between maximal strength values and muscle thickness in the post-test (r = 0.74; *p* < 0.001) were determined but no correlation was found for increases in maximal strength and muscle thickness from pre-to post-test. From this, it can be assumed that maximal strength increases are not related to increases in muscle thickness so that further investigations are required to examine the origin of maximal strength increases. The initial hypothesis can be accepted to a large extent. We examined high interaction effects (ƞ^2^>0.14 and d > 0.8) in the extended knee joint in isometric and dynamic conditions. In both maximum strength tests there were significant increases in maximum strength values in the intervened leg. However, Scheffé test showed no significant differences between maximal strength increases in non-stretched control leg and both legs of the control group. Although the changes in maximal strength of the control leg are not significantly different from the control group under isometric conditions, while Scheffé test showed significant differences between the non-stretched control leg of the intervention group compared to both legs of CG.

In the present work, a stretching duration of 1 hour per day and a weekly volume of 7 hours was realized, which led to comparable results in maximal strength as can be expected from strength training performed two to three times per week (Aube et al., 2020; Pearson et al., 2021). The recorded maximal strength gains can possibly be attributed to muscular adaptations to the mechanical stimuli. A mechanical tension can be seen as an initiating stimulus to induce various cellular processes or signal transduction and induce changes in muscle morphology (Tatsumi, 2010; Mohamad et al., 2011; Riley & van Dyke, 2012; Boppart and Mahmassani, 2019). This so-called mechanotransduction can induce tension-induced muscle hypertrophy (Aguilar-Agon et al., 2019). Smith et al. (1993) and Jacobs & Sciascia (2011) previously showed that stretching tension of sufficient intensity can lead to DOMS and associated inflammation. After this microtraumatization of muscle tissue, the repair processes are related to stimulation of protein synthesis rate (Goldspink & Harridge, 2003; Brentano & Kruel, 2011). Because maximal strength production is closely related to the muscle cross-sectional area of the force-generating muscle, we assume that the muscle tension generated by the one-hour stretching intervention was sufficient to produce muscle hypertrophy and maximal strength gains. We determined muscle thickness *via* ultrasound measurement to investigate structural adaptations of the one-hour stretching training. A similar procedure has already been used by Simpson et al. (2017). The authors investigated the adaptive responses of a three-minute stretching training performed five times per week on maximal strength, muscle thickness, and muscle architecture. Although there were no significant improvements in maximal strength while authors showed muscular hypertrophy (+5.6% in muscle thickness) in addition, Panidi et al. (2021) were also able to determine an enhanced muscle cross-sectional area of 23 ± 14% in the intervention leg vs. 13 ± 14% in the control leg by a 12-week stretching intervention. The cause of the structural change on the control leg seems questionable here due to stretching intervention and possibly are attributed to regular training of the included participants. While central nervous adaptations may be responsible for the contralateral force transfer, which was also recorded in this study, the source of hypertrophic effects on the contralateral leg of 13% must be considered critically, especially since no control group was included in the study. Thus, habituation effects and associated performance gains cannot be ruled out to improve maximal strength production in the non-stretched control leg either.

Another possible explanation for enhanced maximum strength production can be seen in possible changes in muscle architecture, e.g., changes in pennation angle and fascicle length (Cormie et al., 2011a; 2011b). The enhanced maximal strength due to a larger pennation angle is achieved by allowing more sarcomeres to be arranged parallel. In contrast, a higher fascicle length results in optimizing the muscle’s tension-length relationship. While we did not examine muscle architecture and fascicle length, Simpson et al. (2017) found a decrease in pennation angle and an increase in fascicle length in addition to muscle hypertrophy. Normally, a bigger muscle cross sectional area is correlated to an increased pennation angle (Cormie et al., 2011a, 2011b; Suchomel et al., 2018). Consequently, further studies should investigate the influence of long-lasting stretching interventions on muscle architecture as a potential factor for improved maximal strength values. In addition, the changes in muscle architecture recorded by Simpson et al. (2017) suggest an influence on the contraction velocity of the stretched muscle. In addition, study by (Möck et al., 2019) established moderate to high correlations between maximal strength in the calf muscles and sprint performance. Because of achieved significant increase in maximal strength due to one-hour stretching intervention, the influence on sport-specific parameters as jumping and sprinting performance should be investigated in further investigations. Therefore, Panidi et al. (2021) provide first results by recording jumping performance after a twelve-week intervention and examined 27% enhanced vertical jumping heights due to one legged counter movement jump.

While there are studies showing positive effects of stretching interventions on maximal strength (Kokkonen et al., 2007; Nelson et al., 2012; Mizuno, 2019; Yahata et al., 2021) and muscle thickness (Abdel-Aziem & Mohammad, 2012; Moltubakk et al., 2021), there are also studies showing no effects on strength capacity (Sato et al., 2020; Nakamura et al., 2021), hypertrophy and muscle architecture (Nunes et al., 2020; Yahata et al., 2021). Assuming significant influence of stretching intensity on adaptations of the muscle-tendon unit (Apostolopoulos et al., 2015; Nakamura et al., 2021) partially differences in results may be explainable due to heterogeneity in study design of these studies. Most studies did not quantify stretching intensity (Kokkonen et al., 2007; Nelson et al., 2012; Mizuno, 2019) and stretching duration varied to a high degree from 4 × 30 s on 3 days per week (Nelson et al., 2012; Mizuno, 2019) to 6 × 5 min on 2 days per week (Yahata et al., 2021) with very different exercises. Consequently, comparability of results must be questioned and quantification in particular regarding is requested.

Previous studies showing significant increases in maximal strength and/or muscle thickness used shorter stretching duration. Highest stretching volume found in literature was 6 × 5min per session with a weekly volume of 1 h, which was used in our study within 1 day. Compared to Yahata et al. (2021) determining a mean enhancement in maximal isometric strength of 6.4% and 7.8% in maximal dynamic strength with no improvement in muscle thickness, our results show higher increases in maximal strength capacity as well as an improvement in muscle thickness. Considering that we used seven times of the stretch volume compared to Yahata et al. (2021), we demonstrated that increasing the stretching duration leads to increased adaptations as well. Further investigations should examine the most economic stretching duration to improve maximal strength.

Since a contralateral force transfer could be recorded, especially in 1RM measurement, increments in MSt cannot be exclusively attributed to tension-induced hypertrophy effects. After performing intensive strength training, improved distribution of anabolic hormones can be hypothesized, which also have an anabolic effect on the non-stretched calf muscle. However, it seems questionable whether a stretching of the calf muscles of 1 h can result in such a deflection, since especially the amount of hormonal change seems to depend on the size of the involved muscles (Fleck & Kraemer, 2004) and the calf can be considered a relatively small muscle group. In addition to hypertrophy effects in the stretched leg, we hypothesize neuromuscular adaptation through stretching as an additional reason for the effect on maximal strength, since contralateral force transfer due to strength training is also primarily explained by neuromuscular adaptations (Green & Gabriel, 2018; M.; Lee et al., 2009; M.; Lee & Carroll, 2007). Therefore, the inclusion of EMG studies is necessary to clarify neuromuscular adaptations. Since neuromuscular deficits, as well as a loss of muscle mass and cross-sectional area (sarcopenia), lead to reduced balance ability and thus an increased risk of falls (Gschwind et al., 2013; Lacroix et al., 2017), the influence of long-term stretching on balance ability can be investigated in future studies. The calf muscles can be considered relevant, especially in this context (Stolzenberg et al., 2018; Reynoldsid et al., 2020).

Significant improvements in ROM, determined *via* the KtW, were also found to average 13.2% from 12.1 ± 3.0 cm to 13.7 ± 2.6 cm in the intervention leg, while the values for the control leg did not change significantly with −0.8% from 12.7 ± 3.9 cm to 12.6 ± 3.7 cm. ROM values in both control legs measured with KtW did not change significantly. Measurement of ROM by the orthosis revealed a significant improvement of 27.3% in intervened leg from 6.7 ± 1.9 to 8.4 ± 2.0 which corresponds to an angle of 33.5 ± 9.5°–42.5 ± 10°. The contralateral control leg improved flexibility measured *via* the angle measurement device of the orthosis by 7.5% from 6.8 ± 1.9 to 7.2 ± 2.1 with corresponding angle improvement from 34 ± 9.5° to 36 ± 10.5°. No significant changes in ROM could be determined for both legs of the control group.

The influence of stretch training on ROM has already been extensively studied (Medeiros et al., 2016; Medeiros & Martini, 2018). Improvements in ROM in the present study of 13% in the KtW and 27% measured *via* orthosis can possibly be attributed to an increase in serial sarcomere number. In animal experiments, this so-called longitudinal hypertrophy has already been demonstrated by a long-lasting stretch intervention (Antonio et al., 1993; Alway S., 1994, Alway, S. E. 1994). Freitas et al. (2018) and Magnusson (1998)point to an altered pain tolerance at high stretch levels, rather than morphological muscle adaptation, as the cause of expansions in ROM.

Highest effects of stretching the plantar flexors with the orthosis on maximal strength and ROM were determined in testing conditions in extended knee joint. This is explainable as stretching was performed in extended knee joint as well. However, there were significant improvements in maximal strength measured in previous examination of our group and ROM in bended knee joint, too. For listed testing conditions there were significant increases in maximum strength and for 1RM testing significant improvements of the non-stretched control leg. In ROM, no significant effect of the daily 1 h stretching training could be determined in the non-stretched control leg in regard to both control legs.

In conclusion, increases in maximum strength can be commonly attributed to changes in innervation of the central nervous system, changes in muscle architecture or, independently from that, muscle hypertrophy (Loenneke et al., 2019)

### Limitations

Several studies could be found in which ultrasound measurement was used to determine muscle cross-sectional area (Nabavi et al., 2014; Cuellar et al., 2017; Simpson et al., 2017; Messina et al., 2018; Albano et al., 2020; Panidi et al., 2021). In particular, investigating muscle cross-sectional area *via* sonography offers advantages over MRI examinations in terms of cost and time (Sergi et al., 2016). However, stronger or weaker pressure of the ultrasound probe on the muscle belly can influence muscle thickness, so there is a subjective influence on the result. To counteract this, in this study, we took three image acquisitions in succession per leg for each measurement and had the same examiner perform the pretest and posttest of one subject. From a measurement methodology perspective, sonography can be used to investigate structural changes in the muscle, if investigators and evaluators are experienced but the use of MRI images must be considered the gold standard for determining muscle cross-section (Messina et al., 2018; Albano et al., 2020), especially because all subjective factors can be excluded. No randomization could be performed for the present study because not all included subjects agreed to wear the orthosis for 1 h per day.

## Practical Applications

The effects of the training method of long-term stretching on maximal strength, muscle cross-sectional area, and flexibility were investigated in this study can be used in diverse areas. “The therapeutic applications of stretch should therefore be borne in mind when designing regimes for rehabilitation or improved athletic performance” (MacDougall, 2003). Its use in the rehabilitation of orthopedic conditions or lower extremity injuries that result in immobilization seems particularly relevant. A stretching intervention would already be applicable if, due to immobilization or corresponding injuries and diseases, voluntary activation of the musculature in the context of strength training is not (yet) feasible. This could minimize muscle atrophy and loss of strength. Prostheses and cartilage transplants (in the knee and hip) result in long periods of immobilization. This is associated with muscular atrophy (Stevens et al., 2004; Perkin et al., 2016).

## Data Availability

The raw data supporting the conclusions of this article will be made available by the authors, without undue reservation.
