# Supplementary material for: Influence of Long-Lasting Static Stretching on Maximal Strength, Muscle Thickness and Flexibility
Source: Front Physiol. 2022 May 25;13:878955. doi: 10.3389/fphys.2022.878955 (PMC9174468; doi:10.3389/fphys.2022.878955)
Supplement: Supplementary file 1 [file Table1.docx]

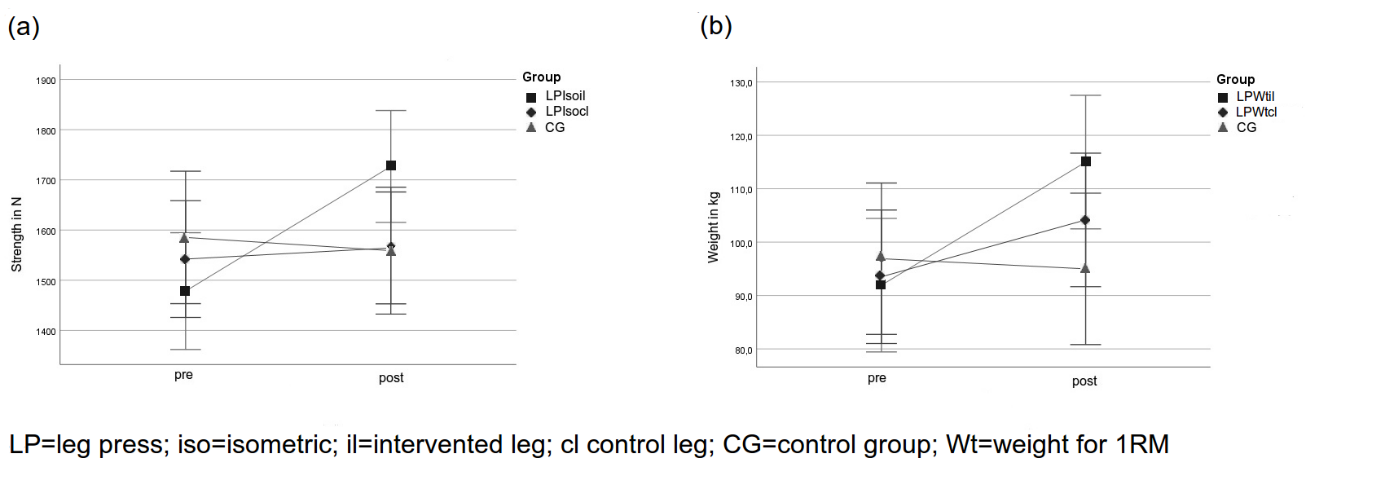


Figure S1: Comparison of maximal strength from pre- to posttest for isometric (a) and dynamic (b) test situation with extended knee. The graph of LPil represents the stretched leg, the graph of LPcl represents the control leg. The Graph in CG represents the right leg of the Control group.


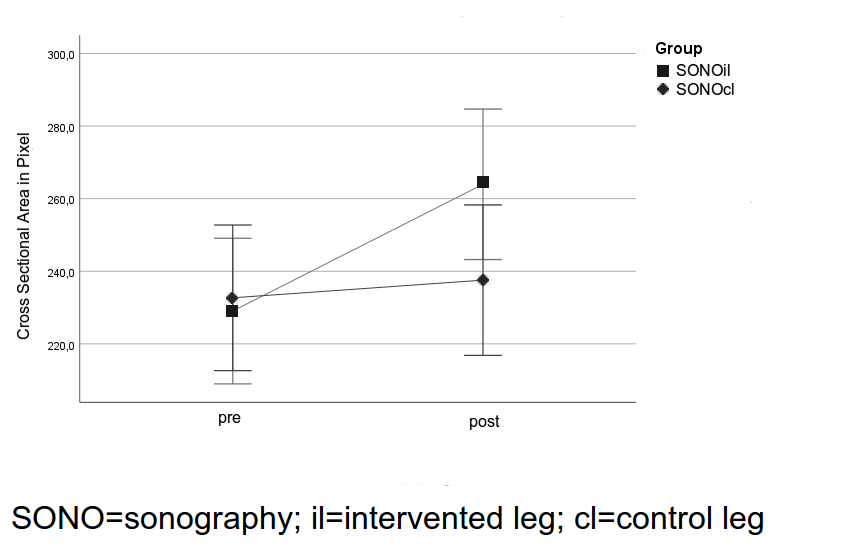


Figure S2: Comparison of muscle thickness measured via Sonography from pre- to post-test between SONOil and SONOcl. The graph of SONOil represents the muscle thickness values of the stretched leg, and the graph of SONOcl represents muscle thickness values of the control leg.


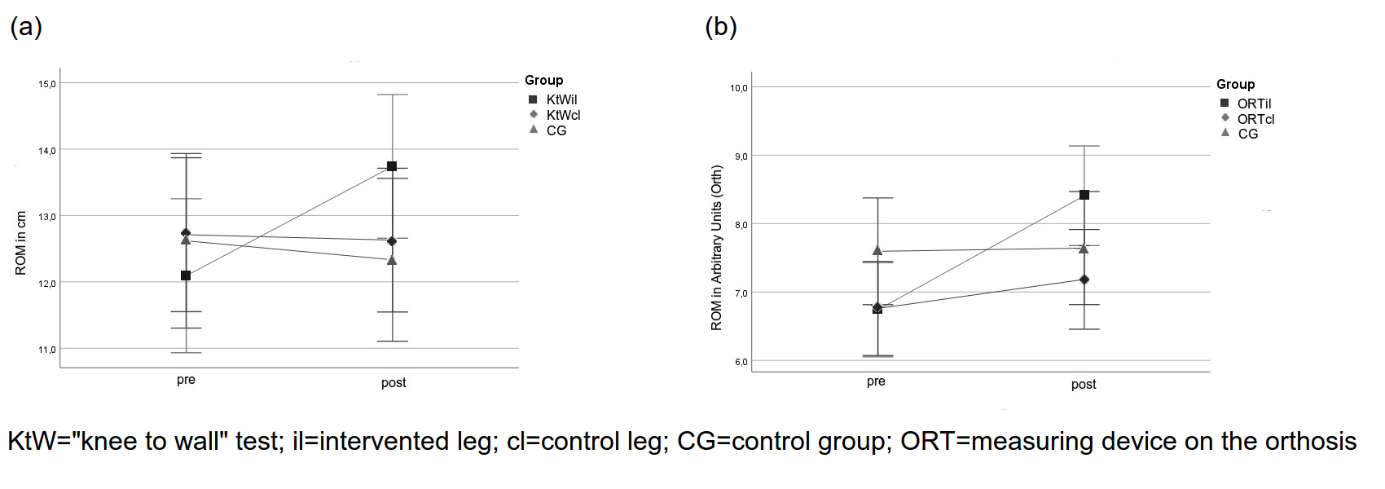


Figure S3: Comparison of ROM from pre- to post-test using KtW (a) and ORT (b) for the stretched and the control leg in IG, as well as the CG.
